# Acute unilateral vestibular neuritis contributes to alterations in vestibular function modulating circumvention around obstacles: A pilot study suggesting a role for vestibular signals in the spatial perception of orientation during circumvention

**DOI:** 10.3389/fnint.2022.807686

**Published:** 2022-10-20

**Authors:** John Allum, Heiko Mario Rust, Flurin Honegger

**Affiliations:** Department of ORL, University Hospital of Basel, Basel, Switzerland

**Keywords:** vestibular loss, object circumvention, vestibular-spinal reflex, spatial orientation, vestibular-ocular reflex

## Abstract

**Background:**

Walking among crowds avoiding colliding with people is described by patients with vestibular disorders as vertigo-inducing. Accurate body motion while circumventing an impeding obstacle in the gait pathway is dependent on an integration of multimodal sensory cues. However, a direct role of vestibular signals in spatial perception of distance or orientation during obstacle circumvention has not been investigated to date.

**Materials and methods:**

We examined trunk yaw motion during circumvention in patients with acute unilateral vestibular loss (aUVL) and compared their results with age-matched healthy controls (HCs). Subjects performed five gait tasks with eyes open two times: walk 6 m in total, but after 3 m, circumvent to the left or right, as closely as possible, a cylindrical obstacle representing a person, and then veer back to the original path; walk 6 m, but after left and right circumvention at 3 m, veer, respectively, to the right, and left 45 deg; and walk 6 m without circumvention. Trunk yaw angular velocities (YAVs) were measured using a gyroscope system.

**Results:**

Yaw angular velocity peak amplitudes approaching to, and departing from, the circumvented object were always greater for patients with aUVL compared to HCs, regardless of whether passing was to the aUVLs’ deficit or normal side. The departing peak YAV was always greater, circa 52 and 87%, than the approaching YAV for HCs when going straight and veering 45 deg (*p* ≤ 0.0006), respectively. For patients with aUVL, departing velocities were marginally greater (12%) than approaching YAVs when going straight (*p* < 0.05) and were only 40% greater when veering 45 deg (*p* = 0.05). The differences in departing YAVs resulted in significantly lower trajectory-end yaw angles for veering trials to the deficit side in patients with aUVL (34 vs. 43 degs in HCs).

**Conclusion:**

The results demonstrate the effects of vestibular loss on yaw velocity control during the three phases of circumvention. First, approaching an obstacle, a greater YAV is found in patients with aUVL. Second, the departing YAV is found to be less than in HCs with respect to the approaching velocity, resulting in larger deficit side passing yaw angles. Third, patients with UVLs show yaw errors returning to the desired trajectory. These results could provide a basis for rehabilitation protocols helping to avoid collisions while walking in crowded spaces.

## Introduction

Walking and navigating among crowds require accurate estimates of one’s own position and angular orientation in space, as well as the associated velocities, relative to those of other people standing or moving nearby (Olivier et al., 2013).

Walking among crowds trying to avoid colliding with people is described by patients with vestibular disorder as vertigo-inducing. This is one particular situation where 45% of patients with chronic vestibular disorders, as reported by several clinical centers, noted difficulties with (Whitney et al., 2016), presumably for three reasons: First, finely controlled trunk yaw angular rotations of the patients and linear distances to the obstacle are required to avoid bumping into someone (Vallis and McFadyen, 2003; Olivier et al., 2013); second, the head and trunk rotate in phase (Vallis and McFadyen, 2003), permitting easier use of the lateral semi-circular canal signals to control trunk velocities; third, compounding the first two reasons because the lateral vestibular semi-circular responses providing yaw control signals are more commonly affected by acute unilateral vestibular neuritis than the vertical canals (Allum and Honegger, 2020a).

It has been suggested that when avoiding an obstacle in the gait path, a new trajectory is accomplished by first turning the head in yaw motion, followed by a yaw and roll motion of the trunk (Patla et al., 1999; Hollands et al., 2001). However, Vallis and McFadyen (2003) found no difference in the onset of trunk and head motion in yaw during circumvention. Furthermore, they found no change in roll motion during circumvention with respect to control trials (no obstacle avoidance). These authors argued that moving the head and trunk segments together simplified the control task for the CNS and reduced the risk of unstable veering behavior. Restricting the degrees of freedom in the yaw plane would have a major advantage for “top-down” sensory integration: First, the peripheral vestibular sensory deficit caused by vestibular neuritis is predominant in the yaw plane (Taylor et al., 2016; Allum and Honegger, 2020a) and would presumably benefit from such a restriction and, second, allow top-down higher order compensation for the dynamic VOR imbalance *via* gaze control of the visual system (Robins and Hollands, 2017). Also, the vestibular deficit could then presumably be more easily compensated for at the level of the brainstem mediated by higher cortical network structures involved in spatial orientation (Lopez et al., 2012; Huang et al., 2015; Kaski et al., 2016), apart from brainstem circuits acting directly to compensate for VOR asymmetries.

**FIGURE 1:**
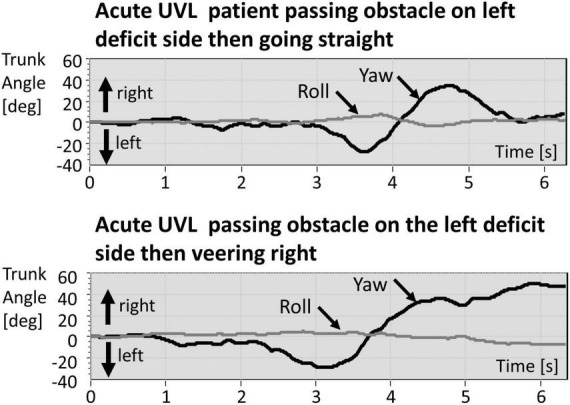
Example traces of trunk yaw and roll of a patient with acute unilateral vestibular loss (aUVL) walking straight **(upper traces)** and veering right **(lower traces)**. The obstacle is passed at approximately 4 s after the start of the recording. Rightward deflections of the yaw traces about a vertical axis perpendicular to the transverse plane are plotted positive, and leftward deflections are plotted negative. Likewise, rightward deflections of the roll traces about horizontal axis perpendicular to the frontal plane are plotted positive, and leftward deflections are plotted negative. Note the amplitudes of roll are smaller than those of yaw angles.

Bearing these sensory integration and central compensation processes in mind, it is noteworthy that the effects that peripheral vestibular loss would have on object circumvention have not been investigated to date. In fact, most investigations into the effect of vestibular loss on postural control have either been restricted to the pitch and roll planes (Carpenter et al., 2001; Matjačić et al., 2001; Allum and Adkin, 2003; Allum et al., 2008; Sienko et al., 2012) or been involved with fixed angular turns in the yaw direction (Glasauer et al., 2002; Péruch et al., 2006), whereas circumvention involves continuous turning in the yaw plane ((Vallis and McFadyen, 2003; Figure 1). Nonetheless, Glasauer et al. (2002) and Péruch et al. (2006) did establish that subjects with vestibular loss had difficulty judging the required turn angle on triangular gait courses when blindfolded. Based on the notion, vestibular loss might provide crucial information on the vestibular-based perception of yaw motion when walking in crowds, we investigated the effect of this loss on circumvention yaw plane motion in patients with acute unilateral vestibular loss by comparing instability of these patients with yaw motion of age-matched healthy control subjects. We specifically included patients with acute vestibular neuritis for the study as these patients generally do not have preceding vestibular problems such as those with a neurectomy to alleviate intractable Meniere’s disease (e.g., see Péruch et al., 2006) or those with a cerebellar pontine angle tumor (e.g., see Glasauer et al., 2002). All three groups of patients do, however, have an ipsi-deficit dynamic vestibular ocular reflex (VOR) loss, which can be identified using video head impulse tests (vHITs), and a static imbalance present as a spontaneous nystagmus (Halmagyi et al., 1990; Taylor et al., 2015; Allum and Honegger, 2020a).

Given that deficits in roll (VOR) responses measured by vHITs are weakly correlated (*R* ≤ 0.55) with deficits in roll postural control and less well correlated for deficits in pitch postural control (Allum and Honegger, 2020b), we were particularly interested in whether, in addition, a new test of vestibulo-spinal function encompassing the yaw direction could result from a study of vestibular loss on circumvention trunk responses. Thus, we also examined, for example, as control conditions, whether yaw plane instability was greater or less than that obtained with walking while rotating the head side-to-side or during normal walking.

**FIGURE 2:**
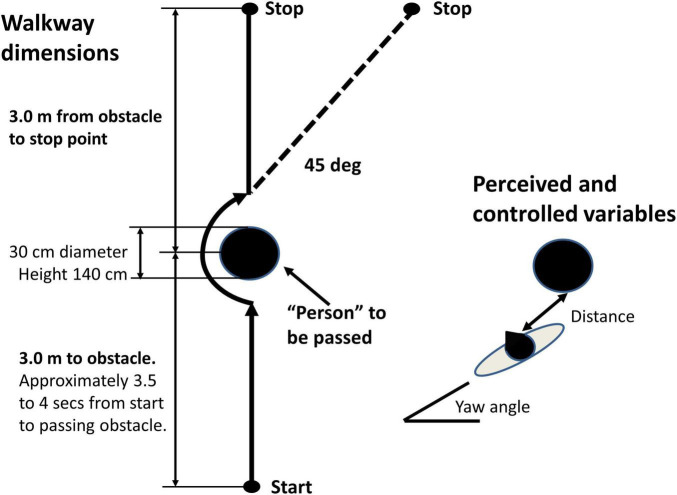
Schema of experimental task. Left walkway dimensions. Right perceived and controlled variables to avoid a collision distance and trunk yaw angle. The subject was requested to walk 3 m toward a cylindrical obstacle, to pass it on the left or right as closely as possible as instructed immediately before the trial, then continue straight, or veer to the right 45 deg, if the obstacle was passed to the left (veering left if the obstacle was passed to right), before stopping 3 m past the obstacle in front of a brick.

It should be borne in mind that the current study of object circumvention (see Figure 2) is a simplified version of the real-life crowd situation, where two persons walking toward one another interact to control the timing and space between themselves when passing one another. Nonetheless, in the real-life and simplified versions, linear and angular measures must be perceived and controlled to avoid a possible collision. Thus, the minimum linear predicted distance between the middle of the shoulders of each person (Olivier et al., 2013) and the yaw angle of the trunk must be controlled so that the shoulders do not touch. This report is focused on yaw angular velocities during circumvention.

## Materials and methods

### Subjects

Patient data collected at the Division of Neuro-otology and Audiology, ORL Clinic, at the University Hospital Basel, were examined retrospectively for this study, which was approved by the Ethics Committee of Northwest and Central Switzerland (EKNZ), approval 2014-026, principal investigator JHJ Allum. A total of four male subjects with a mean age of 60.2 ± 16.4 (±sd) years with acute unilateral peripheral vestibular loss (aUVL) were selected on the basis of a loss greater than 75% for the lateral canal paresis (CP) as measured by caloric testing (mean CP 82.2 ± 7.9%, normal upper limit 30%). The loss was diagnosed as presumably being due to vestibular neuritis (VN) because of the presence of a pathological lateral vHIT gain on the side of canal paresis (mean 0.43 ± 0.13, normal lower limit 0.74, contralateral mean gain 0.85 ± 0.07), the presence of a spontaneous nystagmus (mean at the time of the CP measurements 8.5 ± 3.2°/s) beating toward the healthy ear, nausea, and the constant presence of symptoms over hours. Measurements were taken from caloric testing, vHIT, and balance control trials just after the acute onset of the UVL (on average 3.8 ± 1.1 days after the patient’s diagnosis of VN was established). All the patients were treated intravenously with methylprednisolone (125 mg Solu-Medrol™ per day) and then discharged with oral medication 4–5 days after entry as an in-patient. Data of the patients with aUVL were compared with those of four age-matched healthy controls (HCs), with a mean age of 59.8 ± 17.8 years. Written informed consent was obtained from the patients and HCs for using their data anonymously. Patients with comorbid balance problems due to other causes, for example, peripheral lower leg neuropathy, were excluded from this study.

### Measurement systems

#### Caloric testing

Canal paresis or unilateral weakness was determined using a bithermal (44 and 30°C) caloric test. The differences in average eye slow-phase velocity (SPV) over the culmination phases of nystagmus were compared for the left and right ear irrigations. If R equals the difference between the levels of SPV for the right ear irrigated with 44°C and then with 30°C and L with 30°C, and L the corresponding difference for the left ear, then CP was defined as [(R-L)/(R+L)] × 100%.

#### Video head impulse test

To measure VOR function in response to high angular accelerations (above 2,000°/s^2^), a video head impulse test (vHIT) system was used (ICS system from GN Otometrics, Natus Medical Inc., Taastrup, Denmark). The system was used according to the protocol described by Macdougall et al. (2013), with head angular velocities reaching 100–250°/s by 100 ms. At least 15 head rotations with artifact-free responses in each canal plane were performed.

All vHIT tests were performed by the same person (FH). During the head movements, the patient was seated with gaze fixed on a small target 3 m away. For the vertical canals, the head was first turned 45°, and up or down head rotations were performed in the plane of the canals. Sections of the data with covert saccades and artifacts were removed from the recordings prior to gain calculations by the vHIT manufacturer’s software. Gains were calculated based on the quotient of the areas under the eye and head velocity impulse responses. The interval used started 100 ms prior to peak head velocity and ended when head velocity first crossed zero after this peak.

#### Balance control tests

Participants’ balance control during standard clinical stance and gait trials (Allum and Carpenter, 2005; Hegeman et al., 2007) as well as during object circumvention trials was measured with a SwayStar™ system (Balance International Innovations GmbH, Switzerland). This gyroscope system was attached to the trunk at L1-3 using a converted motorcycle belt. It measured angular velocities in pitch and roll planes for the clinical balance tests. For the circumvention trials, measurements were taken in the yaw and roll planes. Angular displacements were calculated on-line from the measured angular velocities using trapezoid integration. The same standard protocol of 14 stance and gait tasks was used to measure balance control, as described before (Allum and Adkin, 2003). Tasks were performed by the participants without shoes. Stance tasks consisted of standing on one and two legs with eyes open and closed. All stance tasks were ended after 20 s, or when the participant lost balance, or when the non-stance foot touched the ground. Standing on one leg trials were performed on the preferred leg. All stance tasks, except the standing on one leg eyes closed trial, were also repeated on a foam support surface (thickness 10 cm, width 50 cm, length 150 cm, and density 25 kg/m^3^). A semi-stance gait-like task, walking eight tandem steps, was performed on a normal floor and on the foam support system with the participants observing their feet while walking. The following five gait tasks were all performed at the subjects’ preferred gait speed: Three consisted of walking 3 m with either eyes closed, or with eyes open while rotating the head left and right, or while pitching the head up and down. The fourth gait task was to walk over four low barriers, each 24 cm high and spaced 1 m apart. The final task was to walk up and down a set of stairs consisting of two upward and two downward steps, each of height 23 cm.

The circumvention trial procedure is described in Figure 2. The subjects were asked to walk 6 m approaching at 3 m an air-filled obstacle with stabilizing water in its base. The obstacle was 30 cm in diameter and 140 cm in height. The subjects were told that the obstacle represented a person who was to be passed as closely as possible on the left or on the right. On passing the obstacle, the participants were either asked to continue on the same straight trajectory for another 3 m, or veer to the left after passing to the right, or veer to the right after passing to the left (see Figure 2). A brick placed on the floor was used to mark the end of the 6-m trajectory. We used the veering trials to determine if the obstacle departing movement strategy altered the obstacle approaching movement strategy.

During all trials, one or two spotters, as necessary, stood or moved next to the participant to prevent a fall in case of a loss of balance. The duration of each gait trial was the time needed to complete the task or to when the subject lost balance. All balance and circumvention tests were carried out by one of two persons (FH or JHJA).

### Data analysis

As measures of balance control for the clinical stance and gait tasks, we used the peak-to-peak range of angular displacement and velocity in the roll and pitch directions from each trial as well as trial durations. These were combined into a single value, the balance control index (BCI) (Hegeman et al., 2007), as follows:


$$\mathrm{BCI}=2*{\mathrm{s2ecf}}_{\mathrm{pv}}+{\mathrm{tan8}}_{\mathrm{ra}}+1.5*{\mathrm{w3ec}}_{\mathrm{pv}}$$

(1) $$+20*{\mathrm{w3ec}}_{d\,u\,r}+1.5*{\mathrm{w3hp}}_{p\,v}+12*\text{stairs}{}_{r\,a}$$

where s2 stands for standing on 2 legs, ec for eyes closed, f for foam, *pv* for peak-to-peak pitch velocity, tan8 for eight tandem steps, *ra* for peak-to-peak roll angle, w3 for walking 3 m, *dur* for duration, and hp for head pitching.

**FIGURE 3:**
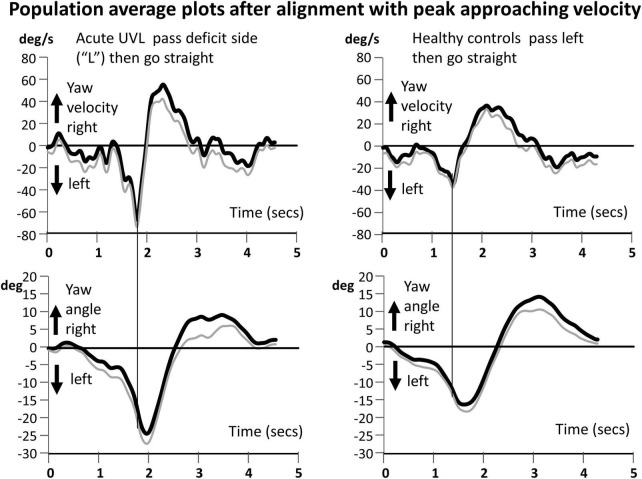
Population yaw angular velocity and yaw angle average plots for the circumvention task of walking to the obstacle, passing it on the acute unilateral vestibular loss side, and then going straight (left traces are for aUVL patients). In the majority of cases, the deficit side was on the left. Therefore, the plots of healthy controls **(right traces)** are shown for passing the obstacle on the left. All traces have been aligned at the peak approaching yaw angular velocity prior to averaging. The population average traces have been filtered with a zero-phase shift second-order Butterworth filter with a cutoff at 5 Hz. The average peak angular velocity and the corresponding time on the yaw angle plots **(lower traces)** are marked by a vertical line. In each panel, the thick black trace is the mean trace (two repetitions of four subjects), and the thin gray line is the mean minus the standard error of the mean (SEM). Note the larger yaw velocities for the subjects with aUVL.

**FIGURE 4:**
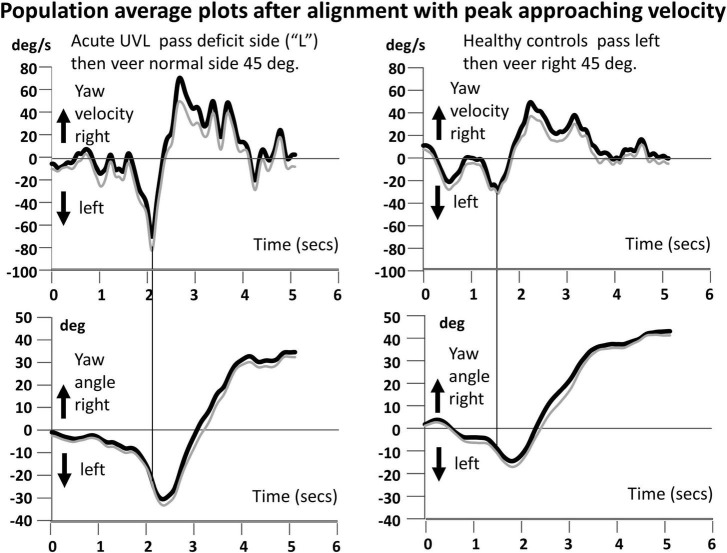
Population average yaw velocity plots for the circumvention task of walking to the obstacle, passing it on the aUVL side, and then veering to the right (left traces). On the right are the traces of the healthy controls. The angle traces in the lower plots show the veering trajectories. Details of the plots are described in the legends to Figures 1, 3.

For the circumvention trials, we measured in addition to peak-to-peak yaw angular velocity, the peak amplitude of approaching yaw velocity as marked by the vertical line in Figures 3, 4, the following peak yaw angle, and the peak departing yaw velocity following the peak yaw angle. The end-of-trial yaw angle was calculated based on last five samples in the trial once trials had been aligned with the peak approaching yaw angular velocity and any offsets at trial onset corrected for. Values from the two identical circumvention trials were averaged together. As three of the four subjects with aUVL had the deficit on the left side, we counted this side as the deficit side and inverted the values of the other patient with a right deficit before computing population averages as in Figures 3, 4 or performing statistical tests of population differences.

We were unable to apply a general linear MANOVA model for repeated measures to the data as there were too few samples. Instead we used univariate ANOVA. For these analyses, participant scores were defined to be dependent on the two fixed effects: two population types (patients with aUVL or HCs) and the four test types [go straight or veer (pass normal or deficit side)]. In order to allow effects to vary across entities, “participants” were set as random effects. For this analysis, R was used (R Core Team, 2020). *Post-hoc* data were compared using parametric *t*-tests in Excel, provided the ANOVA effects were significant (*p* ≤ 0.05). The *post-hoc* analyses described in the “Results” section were corrected for multiple comparisons using a Bonferroni correction.

## Results

### Differences in standard clinical stance and gait tasks

The balance control index (BCI) summary values were significantly different between the populations (*p* = 0.0014). The mean and standard deviation values were 554 ± 44 and 345 ± 16 for patients with aUVL and HCs, respectively. The upper value (95th percentile) of normal BCI values is 460 for persons of the average age of our participants, 60 years.

### Differences in circumvention measures

Circumventing an object leads to characteristic yaw angle and angular velocity profiles, both of which are different depending on whether the subject is asked to continue going straight or asked to veer off to one side after passing the obstacle. As Figure 1 shows, for going straight, the yaw movement is biphasic with the moment of passing by the obstacle corresponding to the zero-crossing of yaw angle motion between the two phases (Figure 1; Vallis and McFadyen, 2003). When the task was to veer off to the opposite direction to that used to pass by the obstacle, a change of motion with respect to going straight was observed just after the second, departing, peak yaw angular motion. The amplitudes of simultaneous roll motion were considerably less than those of yaw motion (see Figure 1; Vallis and McFadyen, 2003) and, therefore, not analyzed in this study.

Population effects were achieved in the ANOVA for both approaching variables, peak velocity and peak angle (*F* > 6.2, *p* < 0.05). A borderline effect was noted for departing peak velocity (*F* = 5.7, *p* = 0.054). There was a test type effect observed for approaching angle (*F* = 5.1, *p* = 0.01) and departing velocity (5.65, *p* = 0.007), but not for approaching velocity.

**FIGURE 5:**
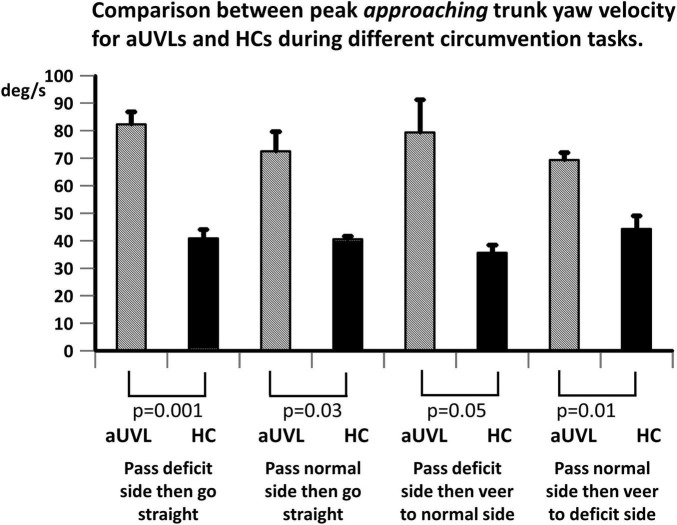
Comparison between mean peak yaw angular velocity approaching toward the obstacle. Peak yaw angular velocities for patients with aUVL and HCs during different circumvention tasks, as listed below the column plots, are displayed. The height of the column represents the mean population value, and the vertical bar, the SEM. The pair-wise levels of significant differences are indicated below the columns.

Our most striking result was a highly significant difference (*p* < 0.001–0.05) in peak approaching YAV between the subjects with aUVL and healthy controls (HCs) across circumvention protocols (see Figures 3–5). Furthermore, for each population, the instruction to veer off to one side, rather than going straight, after passing the obstacle had no influence on the amplitude of the peak yaw angular velocity (YAV) approaching the obstacle (Figures 3–5). Also, there was no difference observed in peak approaching YAV if patients were asked to go around the obstacle to the deficit vs. the non-deficit side (Figure 5).

**FIGURE 6:**
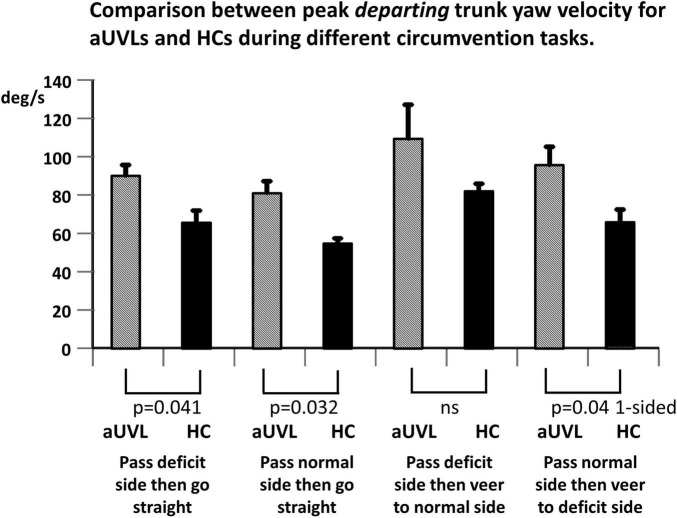
Comparison between mean peak departing from the obstacle peak yaw angular velocities for patients with aUVL and HCs during different circumvention tasks, as listed below the column plots. The height of the column represents the mean population value, and the vertical bar, the SEM. The pair-wise levels of significant differences are indicated below the columns. Note the differences between populations are most significant for the tasks continuing along the same trajectory (no veering).

**FIGURE 7:**
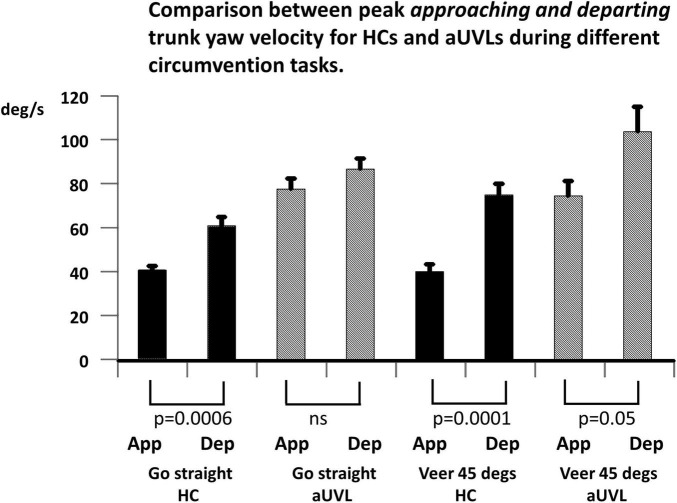
Comparison between mean peak yaw angular velocities approaching toward and departing from the obstacle peak for patients with aUVL and HCs during different circumvention tasks, as listed below the column plots. The height of the column represents the mean population value, and the vertical bar, the SEM. The pair-wise levels of significant differences are indicated below the columns. Note that the HCs show the most significant differences.

Given the aforementioned increase in the YAV of patients with aUVL compared to that in the HCs on approaching the obstacle, two expectations can be formulated for the YAV amplitude of patients with aUVL departing from the obstacle if a stable yaw angle trajectory is to be maintained and the end angles are 0 and 45 degs reached without significant deviation, respectively, for the straight and veered trajectories. First, the departing YAV amplitudes of patients with aUVL should be greater than those of HCs, and second, the ratios of departing to approaching YAVs should be similar to those of HCs; that is, the departing YAVs of UVLs should be proportionally larger than the approaching YAV amplitudes as the departing YAV amplitudes are for approaching YAVs of HCs. Figures 6, 7 illustrate that neither of these conditions are fulfilled. Figure 6 shows that departing aUVL YAV amplitudes in patients are greater than those in HCs but only significantly greater for the straight trajectories (*p* ≤ 0.04). Figure 7 shows that the departing YAVs of HCs are significantly greater than approaching YAVs (*p* ≤ 0.0006) for both the straight and veering tasks with ratios of departing/approaching YAV amplitudes equal to 49.5% and 87%, respectively, whereas the ratios of the UVLs are not significantly greater for the straight trajectories (11.8%) and of borderline significance (*p* = 0.05) for the veered trajectories (39.4%). Despite these differences in the pattern of approaching and departing YAV velocities, the maximum angle deviation of yaw deviation was only significant greater for passing on the deficit side (straight, *p* = 0.05, and veering trajectories, *p* = 0.009). When stopping at the end of the walkway, the end angles only significantly (*p* = 0.04) varied between aUVLs and HCs for the task of passing on the deficit side and veering 45 deg to the normal side [mean end angle aUVLs 34.6 ± 5.4 (sd), HCs 43.0 ± 3.8]. This lack of a general trajectory error suggests that other sensory inputs mostly compensated for the acute vestibular loss after passing the obstacle.

There were no statistical differences between trial durations even though those of the subjects with aUVL tended to be slightly longer. For example, durations for the veering trials were on average 6.05 secs for the HCs and 6.45 for the subjects with aUVL. Over the 6 m of the required trajectory, these gait speeds can be considered as being at preferred and slow gait speeds, respectively (Goutier et al., 2010).

### Comparisons with other gait tests of vestibular function

**FIGURE 8:**
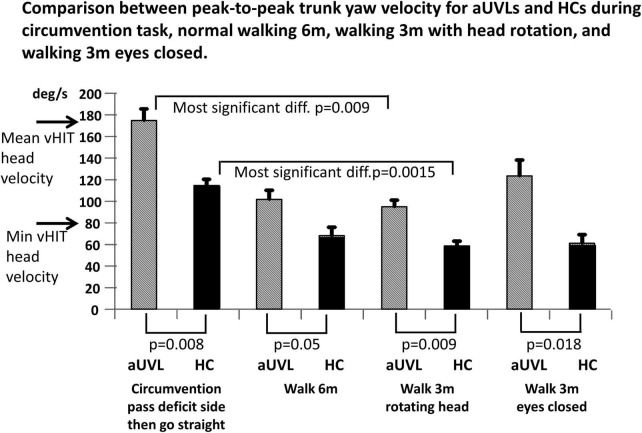
Mean peak-to-peak yaw angular velocities for the circumvention task showing the most significant differences (after Bonferroni correction) between subjects with aUVL and HCs, compared to peak-to-peak differences, for the clinic gait tasks of walking 6 m, walking 3 m with simultaneous head rotations, and walking 3 m with eyes closed. Horizontal arrows on the trunk yaw angular velocity ordinate indicate the minimum acceptable and mean yaw head velocity used for video head impulse tests of the yaw vestibular ocular reflex (data from Cleworth et al., 2017).

Based on the results described earlier, it is possible that the circumvention task also provides a superior test of vestibular influences on gait than currently used clinical tests. Such tests include walking 3 m while rotating the head from side in the yaw plane and walking 3 m with eyes closed (Allum and Adkin, 2003; Allum and Honegger, 2020b). Furthermore, it should be established whether simply walking 6 m provided more significant differences in yaw velocities between patients with aUVLs and HCs than during circumvention tasks. Figure 8 shows that the peak-to-peak YAV amplitudes during the circumvention task of passing on the deficit side then going straight provide the most significant population differences (*p* = 0.008). However, the population differences for the task of walking 3 m while rotating the head from side to side were only slightly less significant (*p* = 0.009). Furthermore, the differences within each population were greatest for the circumvention task, pass deficit side then go straight, compared to the walking task, walking 3 m while rotating the head from side to side (*p* = 0009 for aUVLs, *p* = 0.0015 for HCs). Walking 6 m produced less significant differences between the patients with aUVL and HCs than the circumvention task, as illustrated in Figure 8 (*p* = 0.05 vs. *p* = 0.008).

## Discussion

The current study of object circumvention is a simplified version of the real-life crowd situation where two people walking toward one another interact to control the timing and space between them as they pass one another. The simplified situation is similar to the situation where one of the two people is stationary. In both situations, the minimum predicted distance is controlled to avoid a possible collision (Olivier et al., 2013). In addition, the yaw angle of the trunk must also be controlled. The major simplification we used, as used in previous studies (Vallis and McFadyen, 2003), was to have the stationary person replaced by a model figure towards which subjects were instructed to walk around as closely as possible. Our subjects were those with acute unilateral vestibular loss (aUVL), due to vestibular neuritis, and healthy controls as we were interested in assessing the role of vestibular inputs in this gait task by quantifying how the vestibular dynamic imbalance alters yaw angles and angular velocities during object circumvention. As far as we are aware, this is the first time that vestibular signals have been shown to influence this collision avoidance task. The changed vestibular signals could theoretically be divided into two types, tonic imbalance as measured by the level of spontaneous nystagmus and dynamic imbalance as measured by vHIT gains. Furthermore, the effect of the changed signals could be at two levels, the perception of yaw angles and angular velocities, and the execution of motor commands in yaw. We explore these possibly different effects later. However, it should be borne in mind that our findings are based on preliminary data of few subjects.

### Changes in yaw movement strategies and direct vestibulo-spinal feedback with aUVL

Perhaps the most interesting finding of our study was that the trunk peak yaw velocities of patients with aUVLs approaching the model figure obstacle were equally larger than those of HCs, regardless of whether the object-approaching turning motion of the trunk was to the deficit side or not, and regardless of whether, on passing the obstacle, the desired trajectory was to continue straight or veer off to one side. It is an open question whether the 30–40°/s differences in approaching peak yaw velocities between subject populations are due to an altered perception of yaw velocity due to the level of spontaneous nystagmus or to a reduced vestibulo-spinal feedback gain concomitant with reduced VOR gains seen in vHIT responses.

There are three possible explanations of our preliminary findings that the planned post-obstacle trunk yaw trajectory of aUVLs did not influence the obstacle-approaching peak yaw turning velocity. Either the planned approaching trajectory yaw velocity is set by patients with aUVL to a larger preprogrammed yaw velocity than that set by HCs to ensure a collision does not occur, or, the same velocity is set by the subjects with aUVL as set by HCs, but larger velocities result because the feedback vestibulo-spinal gain countering trunk rotation velocities is too strong, that is, destabilizing, or, third, a combination of the effects occur in patients with aUVL. For the vestibulo-spinal gain explanation to be valid, the equal effect of unilateral vestibular loss on yaw velocities for turning away from the deficit vs. the non-deficit side would be divergent from the clearly asymmetric responses seen in vestibulo-ocular reflexes (Halmagyi et al., 1990; Palla and Straumann, 2004; Allum and Honegger, 2020a). Vestibulo-spinal influences on trunk muscles are inhibitory. Reducing the inhibition leads to a larger muscle response. For pitch plane rotations, the vestibulo-spinal influences on trunk paraspinal and external oblique muscles are laterally equally inhibitory (Carpenter et al., 2001; Allum et al., 2008). Thus, when this influence is absent, increased muscle activity is observed (Carpenter et al., 2001; Allum et al., 2008). Here, we consider the most parsimonious explanation for this common effect on obstacle approaching velocities for all circumvention protocols we used is that the minimum perceived yaw angle and distance to the obstacle to avoid a collision are set larger by the patients with aUVLs; that is, the same strategy (velocity profile) is used by the patients with aUVL and HCs (as shown in Figure 3), but the required amplitude is set larger by patients with aUVLs and is further enhanced by excitatory (dis-inhibited) vestibulo-spinal influences. This is in contrast to the reduced yaw angle executed when patients with a unilateral neuronectomy are asked to complete a triangular gait course (Glasauer et al., 2002; Péruch et al., 2006) possibly because of the continuous yaw angle estimation required with circumvention and also because no possible collision could occur with the triangular course. Future studies should investigate the changes in yaw velocity due to strategy amplitude changes and the changes brought about by dis-inhibited vestibulo-spinal gains.

In order to turn the trunk back to the desired trajectory on passing the obstacle, we expected that the departing peak yaw angular velocity would be greater than the approaching peak yaw velocities for going straight and even larger for the veered trajectories because the yaw motion must be braked and then programmed to move in the opposite direction. This was the case for the HCs whose velocities were 50 and 87% larger, respectively. This was not the case for the patients with aUVL whose departing angular velocities were only 12 and 39% larger, respectively. Again the most parsimonious explanation for this result is that the aUVLs reduced the relative size of the departing angular velocity in order to have a larger minimum perceived obstacle-passing distance. The alternative argument that the dis-inhibited vestibulo-spinal gain that led to instability does not fit with the lowered ratio between departing and approaching velocities observed for the patients with aUVL in comparison to the HCs.

Despite the differences between the yaw velocity characteristics of the patients with aUVL and HCs described earlier, there was little difference between the amplitudes of yaw trajectories at the end of the 6-m walkway, except for the task of walking past the obstacle on the deficit side and then veering 45° to the normal side for which a deviation of 8 deg was observed. Given that approximately 3 s was available to correct the trajectories after passing the obstacle, a variety of sensory inputs, specifically proprioceptive and visual, could be used in this correction process, especially as lower leg proprioceptive, and not vestibular inputs are known to trigger balance corrections (Bloem et al., 2002). Visual inputs, especially those of virtual reality, are known to have a role in modulating circumvention movements (Souza et al., 2018) and balance corrections (Horlings C. et al., 2009). Thus, given the important triggering function of proprioceptive inputs, future experiments should determine the effect of proprioceptive loss on object circumvention particularly after the obstacle is passed.

We measured the yaw motion of the lower trunk at the level of lumbar 1–3, close to the center of gravity. Vallis and McFadyen (2003) measured the upper trunk and head motion and showed that these two segments moved in phase. A weakness of the current study is that head motion was not recorded. Knowing the trajectory characteristics of the head might enable a link to be made between vHIT vestibular ocular responses during head impulse testing and lower trunk yaw angular velocities during circumvention trajectories. Based on previous reports of low-frequency movements (<0.7 Hz) for pitch and roll during stance (Horlings C. G. et al., 2009; Honegger et al., 2012), we would expect that head, and upper and lower trunk yaw motions would also move in phase during circumvention. As can be noted in Figures 1, 3, 4, the yaw angular motion has frequency components predominately below 0.7 Hz.

Regarding ankle and knee joint movements, the question arises if there is a difference in trunk yaw velocities depending on whether the lead leg is the inside or outside leg when passing the obstacle? As we did not measure foot placement, ankle and knee joint motion and, Vallis and McFadyen (2003) did not measure trunk yaw velocities, this question cannot be answered currently. However, it appears from the data of Vallis and McFadyen (2003) that trunk yaw velocity is higher for lead leg inside, rather than outside, even if the amplitude of yaw angle is similar. Thus, future studies should investigate whether the choice of leg to pass the object with is dependent on the side of the side of unilateral vestibular loss and whether this leads to a difference in ankle and knee joint flexion velocities.

### Study limitations and caveats

As mentioned previously, a limitation of the current study is the low number of patients with aUVL tested. This was because we imposed clearly pathological clinical vestibular test results as inclusion criteria. The canal paresis values had to be greater than 75% (the upper normal limit is 30%), the deficit side vHIT gain needed to be less than 0.65 (lower normal limit is 0.74), and the patients needed to be tested within 5 days of diagnosis to minimize the effects of central compensation prior to testing. With these criteria, we obtained clearly significant differences with respect to HCs, as illustrated in Figures 5–8. It should be noted, however, that our results could change with increased numbers of subjects being considered. Furthermore, while power calculations indicated that we had 90% power for the results, as given in Figures 5–8, it should be noted that in case our pilot data were revealing false-positive differences between subjects with vestibular loss and healthy controls, the calculated power would be mistakenly high. Another limitation of this study is the underlying assumption that deficits in vHIT responses in the yaw plane would be directly related to trunk instability in the same plane. As we did not measure head rotations, it is possible that subjects with aUVL rotate the head on the trunk differently from controls during circumvention. Nonetheless, recently, it has been determined that during clinical stance and gait tasks, trunk yaw rotations are strongly correlated (*R* = 0.61) with deficit side vHIT gains of subjects with aUVL (Allum et al., 2022).

### Control of linear distance and trunk yaw angle rotation

It has been emphasized in this report that two variables need to be controlled during obstacle avoidance, the linear distance between the passing person and the obstacle and the yaw angle of the trunk. The former measure was taken as the distance between the midpoint of the shoulders by Olivier et al. (2013). The latter measure, if correctly sensed and programmed, ensures that the shoulders are cleared past the obstacle. In this report, we demonstrated that trunk yaw velocities are altered by acute vestibular loss due to vestibular neuritis with both a tonic and a dynamic imbalance. This result is not surprising, given the greater yaw plane than roll and pitch plane VOR asymmetries (40 vs. 22%) following aUVL due to vestibular neuritis as based on video head impulse test (vHIT) responses (Allum and Honegger, 2020a) and the greater yaw than roll and pitch movements of the trunk during circumvention trials, as we, in this report, and others (Vallis and McFadyen, 2003) have demonstrated. Although we did not measure linear motion of the trunk, we would expect that this would be affected by unilateral vestibular loss because linear accelerations in the transverse plane sensed by the utricles would be affected by the type of vestibular loss our patient participants had. Following the onset of vestibular neuritis, it is common that both the lateral canal (sensing yaw head motion) and utricle responses are affected (Manzari et al., 2011) as these sensory systems are served by the same superior vestibular nerve (Gianoli et al., 2005).

### Use of sensory substitution to aid perception of linear distance and yaw angle rotation

If indeed vestibular and other sensory inputs play an important role in the control of circumvention body movements, the question arises if sensory substitution devices with artificial sensory inputs would be effective in the control of trunk movements during circumvention. There are two ways to approach this question: providing distance information from the obstacle or providing information on trunk angular motion. The first approach appears to work well for visual inputs. When blind individuals were provided with vibro-tactile devices using echo-techniques to indicate the distance to an object to be circumvented, they generally performed better than normal-sighted persons that were blindfolded (Kolarik et al., 2017). According to Kolarik et al. (2017), the better performance of the blind than that of blindfolded sighted participants is consistent with the perceptual enhancement hypothesis that persons with severe visual deficits develop improved auditory abilities to compensate for visual loss. It is also consistent with the evidence that postural control improves when a sound source is present (Anton et al., 2021). The alternative approach providing vibro-tactile feedback of trunk angular motion works well for vestibular loss patients, improving trunk sway in the pitch and roll directions (Honegger et al., 2013; Sienko et al., 2017; Kingma et al., 2019), but it has not been employed for the yaw direction presumably because of coding difficulties with yaw-directed vibro-tactile feedback. Nonetheless, it would be of interest to know which approach vibro-tactile feedback of trunk yaw velocity or obstacle distance or both brought the most perceptual and motor improvement for patients with sensory loss, be it visual, proprioceptive, or vestibular.

### Circumvention as a clinical gait task

Given the significant differences illustrated in Figure 5, it was of interest to us to determine whether a circumvention test could supplant some of the clinical gait tests currently used to differentiate patients with vestibular loss from healthy normal subjects (Allum and Adkin, 2003; Cohen et al., 2012). When developing a new test of vestibular function, a number of criteria should be fulfilled. Most important is that test results should show a clear difference between those of subjects with vestibular loss and healthy, age-matched controls. Ideally, the biomechanical response amplitudes of the trunk should be related to the functional vestibular ocular reflex (VOR) loss, as determined by either high-frequency vHIT responses or low-frequency rotating chair results. Significant correlations have been observed between vHIT roll and yaw response asymmetries and trunk roll and yaw asymmetries, respectively, during various gait tasks (Allum and Honegger, 2020b; Allum et al., 2022). Ideally, the test task should be similar to an everyday task that causes difficulties for subjects with vestibular loss. There are a number of gait tests that have be used to identify the effect of vestibular loss on gait performance (Allum and Adkin, 2003; Cohen et al., 2012). The test most commonly used to induce pathological performance in the yaw plane is walking while rotating the head from left to right (Cohen et al., 2012). For the typical patient with unilateral vestibular neuritis, the range of peak-to-peak yaw trunk velocity is 110 deg/s over 8.5 deg during walking with head rotation compared to 210 deg/s over 63 deg for circumventing an object (see Figure 8). Thus, the possible instability is greater with circumvention than with walking with head rotation. Furthermore, depending on the coupling between the head and trunk, the resulting head motion should be more similar to that imposed on the head during the vHIT (see arrows on the ordinate of Figure 8). During vHITs, motion of the head reaches 150–200 deg/s (Cleworth et al., 2017; Pogson et al., 2019). Thus, the higher yaw velocities present in circumvention trials also provide a better basis for comparing vestibular ocular reflex responses provided by vHIT, with the biomechanical measures of vestibular spinal reflex responses.

## Data availability statement

The raw data supporting the conclusions of this article will be made available by the authors, without undue reservation.

## Ethics statement

The studies involving human participants were reviewed and approved by the Ethics Committee of Northwest and Central Switzerland (EKNZ), approval 2014-026, principal investigator JA. The patients/participants provided their written informed consent to participate in this study.

## Author contributions

FH and JA carried out the data collection and analyzed the results. JA wrote the first draft of the manuscript, which was edited by HR. All authors contributed to the article and approved the submitted version.
